# Therapeutic potential and mechanisms of stem cells in major depressive disorder: a comprehensive review

**DOI:** 10.3389/fphar.2024.1476558

**Published:** 2024-11-25

**Authors:** Jiaqi Li, Yuqian Wang, Yucheng Zhang, Mengran Liu, Xinao Rong, Jinlan Jiang

**Affiliations:** Scientific Research Center, China-Japan Union Hospital, Jilin University, Changchun, China

**Keywords:** depression, stem cells, mechanisms, immunomodulation, review

## Abstract

Depression is a common affective disorder characterized by persistent low mood, diminished interest or pleasure in normally enjoyable activities, disturbances in sleep patterns, and suicidal ideation. Conventional treatments often yield unsatisfactory results and are associated with several adverse effects. However, emerging literature has highlighted the potential of stem cell (SC) transplantation as a promising avenue for treating depression owing to its favorable anti-inflammatory and neurotrophic properties. This review summarizes the therapeutic effects and underlying mechanisms associated with SC transplantation in depression, offering a conceptual framework for the future application of SCs in the clinical treatment of depression.

## 1 Introduction

Depression is a prevalent affective disorder worldwide. According to statistics from the World Health Organization, approximately 322 million people suffer from depression, accounting for 4.4% of the global population. It ranks third among global diseases and is projected to become the second most prevalent by 2030, imposing a significant socioeconomic burden (Malhi and Mann, 2018). Depression is characterized by high recurrence and suicide rates, along with significant social dysfunction among patients. Severe cases may even involve hallucinations, violence, and suicidal tendencies, posing serious threats to both the individuals and others (Health Quality Ontario, 2017; Copeland et al., 2021). In addition, depression commonly coexists with serious chronic diseases such as diabetes, hypertension, and cancer, exacerbating their symptoms and hindering treatment (Cui et al., 2024).

Currently, the primary treatments for depression include psychological counseling and medication (Hillhouse and Porter, 2015). While psychotherapy is widely utilized, it has various shortcomings such as the lack of standardized protocols, limited long-term follow-up, and sample size limitations. Medications, including tricyclic antidepressants (TCAs), selective serotonin reuptake inhibitors (SSRIs), and monoamine oxidase inhibitors (MAOIs), are commonly prescribed (Cleare et al., 2015; Lane, 2015; Purgato et al., 2015). However, these drugs often entail side effects such as drowsiness, cardiac QT interval prolongation, and sexual dysfunction. Additionally, around one-third of patients with refractory depression do not respond well to these drugs. The lack of validated clinical tests has impeded a complete understanding of depression’s pathogenesis, necessitating the development of new antidepressant treatments.

Stem cell (SC) transplantation, a cutting-edge therapeutic approach, holds promise across various medical fields, including hematological disorders, neurodegenerative diseases, and certain cancers. Notably, it is increasingly being studied for its potential in addressing depression. For example, a recent clinical trial involving 16 patients with depression who underwent SC transplantation therapy reported improvement in all patients and decreased Beck’s scores within 45 days post transplantation (Smulevich et al., 2016). Numerous other studies have also highlighted the potential antidepressant effects of SC transplantation, underscoring its therapeutic potential for depression.

Depression substantially diminishes patient’s quality of life, interpersonal interactions, and work capability, imposing a significant burden on society. Therefore, early intervention and timely treatment are crucial. SC transplantation, as an emerging therapeutic method, can promote neuronal growth, repair damaged neural networks, and regulate neurotransmitter balance thereby improving mood and psychological wellbeing. This review aimed to explore the relationship between SC transplantation and depression, analyze the mechanisms underlying the development of depression, elucidate the effects and mechanisms of SC transplantation intervention in depression, and provide a theoretical framework for applying SC transplantation in depression treatment.

## 2 Pathophysiology of depression

Depression manifests as a clinical syndrome characterized by significantly low mood; with its underlying causes being complex and not fully understood. Researchers have proposed various mechanisms for the pathogenesis of depression, including the hypothalamic-pituitary-adrenal axis (HPA) dysfunction hypothesis, monoamine hypothesis, inflammatory hypothesis, genetic and epigenetic abnormality hypothesis, structural and functional brain remodeling hypotheses, and psychosocial hypothesis (Stetler and Miller, 2011; Malhi and Mann, 2018; Milaneschi et al., 2020; Li et al., 2021).

### 2.1 Monoamine hypothesis

A biological mechanism of depression may involve the deficiency of monoamines, specifically 5-hydroxytryptamine (5-HT), norepinephrine (NE), and dopamine (DA) (Hamon and Blier, 2013). While medications such as MAOIs and TCAs may ameliorate depressive symptoms by enhancing the activities of 5-HT and NE, this hypothesis faces challenges, as the clinical effects of antidepressant medications typically takes weeks to manifest, despite their ability to increase monoamine levels almost instantly. Furthermore, approximately one-third of patients do not respond to antidepressants that solely inhibit monoamine reuptake, and limiting the availability of the 5-HT precursor tryptophan does not induce depressive episodes in all individuals (Kaltenboeck and Harmer, 2018). Consequently, monoamine deficiency may not be universally present in all patients, suggesting the involvement of alternative pathways in depression’s pathophysiology.

### 2.2 Neuroinflammatory hypothesis

#### 2.2.1 Oxidative stress

Numerous studies have identified mitochondrial dysfunction in certain individuals with depression, characterized by altered mitochondrial structure and function, including decreased ATP production. This dysfunction is accompanied by the generation of free radicals and oxidative stress, which escalate as the disease progresses (Singh et al., 2019). While reactive oxygen species (ROS) at normal levels serve as important signaling molecules in neuronal function, excessive ROS in the presence of low antioxidant levels can be harmful to neurons. Heightened oxidative stress may exacerbate mitochondrial damage, leading to increased apoptosis and the release of inflammatory signals, thereby damaging cells in the brain (Sies and Jones, 2020). Overall, mitochondrial dysfunction may trigger the activation of apoptosis, releasing damage-associated molecular patterns and amplifying inflammatory mechanisms. The resulting oxidative stress may contribute to the accelerated cellular senescence observed in patients with depression.

#### 2.2.2 Cytokines and microglia differentiation

Mounting evidence indicates the pivotal role of neuroimmunity in mood disorder development and remission (Koo and Wohleb, 2021). Microglia, among the central nervous system’s (CNS) most diverse cell types, exhibit heterogeneity across developmental stages, colonization sites, and disease states. They perform various functions, including phagocytosis of cellular debris, promotion of neurogenesis, and synaptic pruning, as well as release cytokines and chemokines (Tan et al., 2020; Ceasrine and Bilbo, 2021). Microglial activation, traditionally categorized as neurotoxic (M1-phenotype microglia) or neuroprotective (M2-phenotype microglia), is heterogeneous. Microglia are divided into the M1 (classical activation) and M2 (alternative activation) phenotypes based on their activation status (Glass et al., 2010; Luo and Chen, 2012). M1 microglia, expressing proinflammatory cytokines (tumor necrosis factor-α [TNF-α], interleukin [IL]-1β, IL-6, inducible nitric oxide synthase [iNOS], and CCL2), may contribute to neurotoxic outcomes by disrupting the neurotrophic system (Kwon and Koh, 2020). Conversely, M2-phenotype microglia release different mediators (Ym1, Arg1, IL-10, IL-4, and TGF-β) to counteract inflammation-induced CNS damage (Liu et al., 2020). Pathological studies have observed increased expression of TNF-α, IL, IL-1β, IL-6, and C-X3-C modality chemokine receptor 1 (CX3CR1) in the brain of patients with depression (Rahimian et al., 2021). Studies have also reported a significant increase in mRNA expression of Toll-like receptors (TLRs) TLR3, TLR4, and TLR7 in the prefrontal cortex of individuals with depression, suggesting microglial activation toward a proinflammatory state and an enhanced inflammatory response (Pandey et al., 2019). Additionally, heightened microglia activation has been observed in the white matter region of the dorsal anterior cingulate gyrus in suicidal patients with depression, although the expression levels of IL-1β, TNF-α, and IL-10 were not elevated (Torres-Platas et al., 2014).

#### 2.2.3 Immune microenvironment

T helper 17 cells (Th17) play a crucial role in regulatory T-cell (Treg) development and function, with the balance between these cell types crucial for immune homeostasis (Cui et al., 2021). Under normal physiological conditions, Th17 cells regulate hippocampal growth and maintain the structural integrity of the brain without detrimental effects on the CNS. However, in individuals with depression, peripheral T cells exhibit poor adaptability to the proinflammatory environment, correlating positively with Th17 cells (Davami et al., 2016). Patients with depression show elevated levels of IL-17 and reduced Treg cell numbers, leading to an increased Th17/Treg ratio. Furthermore, a study encompassing 33 cohort or case-control studies examined changes in absolute versus relative peripheral blood immune cell counts between individuals with depression and matched controls. A meta-analysis of 27 of these studies, involving 1,286 patients with depression and 991 controls, found that compared with controls, patients with depression have significantly higher absolute counts of CD4^+^ T cells, significantly lower relative counts of Th1 and Th2, and significantly higher mean absolute counts of B lymphocytes (Foley É et al., 2023). Taken together, the bidirectional communication network between the CNS and immune system plays a pivotal role in the pathological process of depression, with both acute and chronic stress contributing to the development of depression through neuroimmune mechanisms.

### 2.3 Neurotrophic factor and hippocampal neuron hypothesis

Neurotrophic factors (NTF) are peptides or small proteins crucial for neuronal growth, differentiation, and survival, playing vital roles in brain development, homeostasis, and plasticity (Huang and Reichardt, 2003). Among them, brain-derived neurotrophic factor (BDNF) stands out as a key mediator of neuronal synaptic plasticity in the brain (Gonul et al., 2005). BDNF exerts significant effects on neuronal morphology and physiology, promoting neuronal growth, synapse formation, and stabilization in brain nerve cells (Zhang and Liao, 2020). As a vital member of the neurotrophic factor family, BDNF activates tropomyosin receptor kinase (Trk) and the p75 receptor. Studies have shown that insufficient BDNF secretion can impede the continued differentiation of immature neurons and greatly reduce synaptic plasticity, neurotransmission, and receptor sensitivity in mature neurons, leading to progressive nerve damage and depression onset. Studies have shown significantly lower levels of BDNF protein and mRNA and reduced mRNA levels of its receptor TrkB in the brain of patients with depression compared with those in the brain of healthy individuals (Dwivedi, 2013). Moreover, the amount of phosphorylated TrkB in the active state is also reduced (Castrén and Monteggia, 2021). BDNF is implicated in spontaneous activity-independent synaptic transmission, contributing to a novel form of plasticity associated with the rapid antidepressant effects of ketamine (Autry et al., 2011; Nosyreva et al., 2013; Gideons et al., 2014). Low expression of BDNF or TrkB in broad forebrain regions inhibits the antidepressant-like behavioral effects of ketamine and potentiation of hippocampal synapses in mice (Björkholm and Monteggia, 2016). The hippocampus, a crucial part of the limbic system, plays an important role in consolidating short-term, long-term, and spatial memories. Hippocampal neurons are stress-sensitive, and imaging studies have shown that the density of the hippocampal region in patients with depression is decreased (Cobb et al., 2013). Additionally, neuronal dendrites show reduced complexity and increased necrosis, possibly indicating permanent impairment of hippocampal neuroplasticity (Amico et al., 2011). Reduced hippocampal neurogenesis has been linked to depression-like behaviors in animals (Snyder et al., 2011), and the efficacy of antidepressant treatment largely depends on improvements in hippocampal neurogenesis (Mateus-Pinheiro et al., 2013). A study by Lupien et al. found that reduced hippocampal volume and memory deficits were associated with elevated cortisol levels. Moreover, Banasr et al. found that hippocampal neurogenesis is related to 5-HT levels and can be modulated by various common 5-HT receptor subtypes (Berhan and Barker, 2014).

### 2.4 HPA dysfunction hypothesis

The HPA plays a pivotal role in coordinating the body’s response to stress. Upon encountering physiological or psychological stressors, the HPA is activated, initiating a hormonal cascade. Signals from tissues such as the hippocampus prompt the release of corticotropin-releasing hormone (CRH) from the paraventricular nucleus of the hypothalamus, leading to the secretion of adrenocorticotropic hormone from the anterior pituitary gland, and ultimately resulting in the release of glucocorticoids (GCs) from the adrenal gland (Asarnow, 2020). However, persistent external stress can lead to excessive GC secretion and the dysregulation of feedback systems in the body (Canet et al., 2018). The primary GC secreted is corticosterone (CORT), and elevated CORT levels are an important mechanism underlying depression induced by CNS damage. Patients with depression often exhibit elevated CORT levels, HPA overactivity, and dysregulation of HPA feedback mechanisms. Consequently, various drugs targeting the HPA have been developed to treat depression, including corticosteroid synthesis inhibitors, GC receptor antagonists, and CRH receptor antagonists.

### 2.5 Genetic and environmental factors in depression

The pathogenesis of depression is intricately linked to both environmental and genetic factors. Data indicates that the probability of depression in relatives of patients with depression is approximately 10–30 times that of the general population, and the closer the blood relationship, the higher the probability of the disease. Patients with depression often exhibit altered brain structures, characterized by reduced glial density in regions such as the prefrontal cortex, hippocampus, and amygdala. In addition, exposure to stressful events, such as changes in environmental and personal circumstances, can serve as significant triggers of depression onset.

## 3 Potential role and mechanism of SCs in the treatment of depression

SCs represent a versatile class of cells capable of replication and possessing multidirectional differentiation potential. Under specific conditions, SCs can differentiate into a variety of cells that form various tissues and organs in the human body. Notably, SCs exhibit four key properties: self-renewal, multidirectional differentiation, homing ability, and low immunogenicity. They have emerged as promising candidates for the treatment of numerous neurological disorders (Laplane and Solary, 2019). In recent years, SCs have been successfully used to treat neurological disorders, such as Alzheimer’s and Parkinson’s diseases. Studies have confirmed a significant increase in NTF release following SC transplantation for neurodegenerative diseases. This underscores SCs capacity to improve and repair the structure and function of the nervous system to a certain extent (Enciu et al., 2011; Park et al., 2015; Jalali et al., 2020), laying a foundation for exploring SC therapy for the treatment of depression. Several studies have shown that SCs not only differentiate into neurons to modulate neurotransmission dysfunction but also release NTFs, enhancing neurotransmission function and ameliorating neurological damage through paracrine mechanisms. Additionally, SC transplantation prompts the release of a large number of anti-inflammatory factors, inhibiting oxidative stress and fostering an improved inflammatory microenvironment within the brain (Table 1).

**TABLE 1:** Application of mesenchymal SC (MSC) in major depressive disorder.

Sources of SCs	Animal model	Mechanisms	References
Bone marrow	Flinder Sensitive Line rats (FSLs), a genetic model of depression	Stimulation of increased mRNA and protein levels of BDNF	Salem et al. (2018)
Bone marrow	Flinder Sensitive Line rats	Increasing NTF that promotes neurogenesis	Tan et al. (2020)
Bone marrow	Mouse model of depression induced by chronic restraint stress (CRS)	Activation of dorsal raphe nucleus (DRN) 5-hydroxytryptamine (5-HT) neurons and activation of vagal sensory neurons innervating the pulmonary system	Huang et al. (2020)
Adipose tissue	Mouse model of depression induced by chronic mild stress (CMS)	Downregulation of inflammatory factors such as IL-6, IL-1, TNF-α, TLR-4, NF-κB, and MCP-1	Huang et al. (2023)
Human umbilical cord	Sprague Dawley rat model of depression induced by LPS injection and chronic unpredictable mild stress (CUMS)	Increasing expression of anti-inflammatory cytokines	Wang et al. (2024)
Human umbilical cord	Mouse model of depression induced by CUMS	Decreasing levels of proinflammatory factors and increasing levels of anti-inflammatory factors; inhibition of microglial M1 polarization and inflammatory factor levels; reduction of neuronal damage	Laplane and Solary (2019)
Bone marrow	Mouse model of depression induced by CMS	Reduced expression of inflammatory factors in the serum and reduced activation of hippocampal microglia	Huang et al. (2023)
Bone marrow	Sprague Dawley rat model of depression induced by corticosterone injection	Upregulation of miR-26a which ameliorates hippocampal neuronal damage	Guo et al. (2020)
Human umbilical cord	Mouse model of depression induced by myocardial infarction	Promoting the transformation of M1 microglia into the M2 phenotype	Zhang et al. (2021)

### 3.1 MCs activate 5-HT neurons within the dorsal raphe nucleus (DRN) to alleviate depressive-like behavior

Under the influence of various pathological factors such as infection, stress, and metabolic disorders, excessive activation of microglia can elevate the activity of indoleamine 2, 3-dioxygenase (IDO), the rate-limiting enzyme in tryptophan catabolism. This process reduces the concentration of tryptophan, the precursor for 5-HT synthesis, thereby decreasing neurotransmitter levels in neurons. Recent studies have shown that mesenchymal SCs (MSCs) can be injected into the nucleus of the solitary tract through the BDNF-TrkB signaling pathway to induce the release and activation of 5-HT in the DRN thereby exerting anti-anxiety and anti-depression effects (Huang et al., 2023). In addition, exosomes secreted by MSCs have been found to enhance the expression of β-endorphin and the synthesis and secretion of 5-HT and DA in the brain. This augmentation of neurotransmitter levels improves brain excitability and alleviates depressive symptoms. Huang et al. demonstrated significant neural activation within the DRN serotonergic system following MSC injection (Huang et al., 2023). *In vivo* electrophysiological and patch-clamp recordings of the DRN region confirmed the activation of serotonergic neurons by intravenous infusion of MSCs. Studies have further indicated that MSCs exert their effects directly on sensory neurons through paracrine mechanisms, transmitting signals to the CNS. Intravenous MSC administration thus exerts antidepressant and anxiolytic effects in mice by regulating the remote CNS serotonergic system through the pulmonary vagal-brain axis. This research unveils a functional link between the lungs and brain via the pulmonary vagus nerve, inspiring exploration into the multifaceted potential mechanisms of MSC action in depression treatment.

### 3.2 SCs improve depression-like behavior through suppression of neuroinflammation

#### 3.2.1 SCs improve depressive behavior by inhibiting oxidative stress *in vivo*

Evidence suggests that the brains of patients with depression have a lower antioxidant capacity and higher oxidative damage than those of healthy controls. Mitochondrial dysfunction, often associated with depression and other neuropsychiatric disorders, serves as a significant source of oxidative damage. In addition, neuroinflammation, prevalent in the brains of patients with depression, contributes significantly to ROS production (Riveros et al., 2022). MSCs have been shown to reduce inflammation and oxidative stress in numerous diseases, including colitis, pancreatitis, arthritis, sepsis, stroke, myocardial infarction, and hyperoxic lung injury (Stavely and Nurgali, 2020). Hydrogen sulfide (H2S), known for its potent anti-inflammatory and antioxidant properties, exhibits reduced plasma levels in patients with depression and directly correlates with the severity of depression (Yang et al., 2021). MSCs possess the ability to generate H2S, which is essential for maintaining MSCs function and increasing their survival and proliferation under inflammatory and oxidative conditions (Yang et al., 2016). Furthermore, H2S has been demonstrated to upregulate Sirt1 expression and alleviate depression-like symptoms induced by sleep deprivation in rats, attenuating levels of proinflammatory cytokines (IL-1β, IL-6, TNF-α, and CCL2), and augmenting anti-inflammatory cytokines in the hippocampus (Kang et al., 2021). Several studies have indicated that MSCs increase the expression of anti-inflammatory factors, enhance antioxidant defenses, and improve the behavioral manifestations of depression (Salem et al., 2018; Cui et al., 2019; Wang et al., 2024). However, no reports directly link MSCs to ROS or mitochondrial function modulation for improving depressive symptoms. Due to the complexity of the brain microenvironment, the precise mechanism by which MSCs protect patients with depression from inflammation-induced oxidative stress remains to be explored.

#### 3.2.2 SCs improve depressive symptoms by modulating proinflammatory factors

Cytokines, including interleukins, tumor necrosis factors, and interferons, are important regulators of depression-associated inflammation. Prior research suggests that the implantation of SCs and their secretory properties contribute to the production and downregulation of a large number of anti-inflammatory factors (Cui et al., 2019), exerting anti-inflammatory and neuroprotective effects akin to antidepressants. For example Salem et al. (2018), bone marrow-derived MSCs (BMSCs) ameliorate brain inflammation by decreasing immunoreactivity to insulin growth factor-1 receptor, synaptophysin, and cachectomycin-3, thereby mitigating inflammation and oxidative damage. Furthermore, SC transplantation improves the inflammatory environment of the brain by downregulating the levels of inflammatory factors such as IL-6, IL-1 and upregulating the anti-inflammatory factor IL- 10. In a recent study by Wang et al., using a rat model of depression induced by chronic unpredictable mild stress and lipopolysaccharide (LPS) co-stimulation, human umbilical cord blood MSC (hUC-MSC) treatment led to decreased expression of proinflammatory cytokines, such as IL-6 and TNF-α, and increased expression of anti-inflammatory cytokines, such as IL-10 and TNF-β. Notably, a unique feature of this study was that rats with depression reliably maintained their depressive behavior owing to continuous LPS administration during hUC-MSC treatment. The study demonstrated that hUC-MSC treatment induced the expression of anti-inflammatory cytokines, effectively counteracting LPS-induced hippocampal injury (Wang et al., 2024).

#### 3.2.3 SCs improve depression-like behavior by shifting microglial phenotype from M1-to M2-type

Microglia, resident immune cells of the CNS, play a key role in maintaining brain homeostasis. Overactivation of microglia is implicated in the pathogenesis of depression (Aguzzi et al., 2013), with activated microglia typically categorized into the M1 and M2 types. M1 microglia, through the expression of proinflammatory cytokines, can induce neurotrophic system dysfunction, leading to neurotoxicity (Kwon and Koh, 2020). Conversely, M2 microglia, considered the neuroprotective phenotype, release various mediators (Ym1, Arg1, IL-10, IL-4, and TGF- β) to counteract inflammation-induced CNS damage (Liu et al., 2020). A growing body of evidence suggests that neuroinflammation mediated by M1-type polarized microglia plays a key role in the pathogenesis of depression. MSCs possess immunomodulatory properties that induce reprogramming of microglia from a proinflammatory (M1) to an anti-inflammatory (M2) phenotype (Li et al., 2020; Liu et al., 2021). In a rat model of subarachnoid hemorrhage, immunomodulatory MSCs promoted the differentiation of microglial cells into the anti-inflammatory M2 phenotype, significantly improving the inflammatory environment in the brain (Na et al., 2020). This “calming” effect may involve the chemokine axis CX3CR1/CX3CL1, released by activated MSCs, which upregulates the expression of the CX3CR1/CX3CL1 axis, inhibits microglial activation, and converts activated cells to a quiescent state. SC-derived exosomes (Exos) exhibit immunomodulatory functions; in a model of cerebral ischemia/reperfusion injury, BMSC-Exos induced a shift from M1-type polarized microglia to the M2 phenotype. *In vitro* models confirmed that BMSC-Exos attenuate neuronal death by modulating microglial polarization. Moreover, Li et al. confirmed that in a mouse model of chronic unpredictable stress-induced depression, hUC-MSCs altered the polarization of microglia to improve depressive symptoms by reducing the activation of complement C3a-C3aR signaling (Li et al., 2020).

#### 3.2.4 SCs remodel the immune microenvironment by regulating other immune cells

Studies have shown that patients with depression have increased numbers of natural (neutrophils and monocytes) and adaptive immune cells (Th1, Th17, and plasma cells) in their peripheral circulatory system (Drevets et al., 2022). In addition, a recent study established, for the first time, a link between depression and the mechanical characteristics of all major blood cell types. This study showed that depression, especially persistent depression lasting more than 2 years, is associated with increased blood cell deformability. Although all major blood cells tended to exhibit increased deformability, lymphocytes, monocytes, and neutrophils were the most affected. This may indicate deterioration in cellular function and further explain the generalized lethargy observed in many patients with depression (Walther et al., 2022). MSCs exhibit immunomodulatory functions, capable of enhancing or inhibiting immune cell proliferation and responses, thus influencing immune reconstitution.

The immunosuppressive mechanism of SCs mainly regulates a variety of immune cells by secreting IDO, TGF-β, NO and other factors (Li et al., 2023). MSCs have been found to maintain macrophages and dendritic cells in an immature or anti-inflammatory state, thereby preventing the activation of effector T cells and promoting the production of Tregs (Gao et al., 2016; Jiang and Xu, 2020). BMSCs acquire T-cell suppressive properties in the presence of IFN-γ produced by CD4^+^ helper T cells and cytotoxic CD8^+^ T lymphocytes (Rivera-Cruz et al., 2017). Huc-MSCs inhibit T lymphocyte proliferation, downregulate RORγt mRNA and protein expression, decrease the proportion of Th17 cells, and increase the proportion of Tregs in the spleen. Furthermore, they downregulate RORγt and Foxp3 expression in the joints. Nevertheless, research elucidating the mechanism of MSC-mediated immune microenvironment remodeling in depression models remains limited. Despite a growing body of research on the immunomodulatory effects of MSCs and depression, the precise molecular mechanisms underlying their interactions require further study.

### 3.3 SCs promote NTF expression neurogenesis

NTF plays a key role in neuroplasticity, and reduced NTF levels along with decreased neurotransmitter concentrations can lead to the onset of depression. Studies in animals and limited clinical studies suggest that MSCs ameliorate depressive symptoms by releasing NTF, exerting paracrine effects. A recent study by Huang et al. further validated the therapeutic potential of MSC transplantation for depression via its anti-inflammatory effects (Huang et al., 2020). Employing adipose-derived MSCs (ADSCs), the study injected C57BL/6 mice with ADSCs during the chronic mild stress protocol. Behavioral tests revealed that ADSC treatment upregulated BDNF expression and its receptor TrkB in brain tissue, effectively alleviating depression-like behaviors in mice. Similarly, Kin et al. demonstrated antidepressant effects in Wistar Kyoto rats through the neurogenic pathway by implanting encapsulated MSCs (eMSCs) into the lateral ventricle (Kin et al., 2020). The eMSCs inhibited depression-like behavior and enhanced endogenous neurogenesis in the subventricular zone and dentate gyrus of the hippocampus, primarily through the stable secretion of growth factors such as vascular endothelial growth factor, BDNF, fibroblast growth factor 2, and ciliary neurotrophic factor. Moreover, intravenously injected MSC were shown to secrete BDNF, activating vagal sensory fibers in the lungs and transmitting signals to the CNS through the pulmonary vagus-solitary-dorsal raphe pathway, thereby ameliorating depressive- and anxiety-like behaviors in mice (Huang et al., 2023).

Human embryonic SCs (ESCs) and human induced pluripotent SCs (iPSCs) possess the ability to differentiate into all somatic cell types in the human body. Pre-neural transcription factors can induce the generation of neuronal cells from ESCs and iPSCs, facilitating large-scale studies on human neural differentiations and neuropsychiatric diseases (Liu et al., 2023). Guiding the differentiation of SCs into neurons and implanting them into the brain has been shown to promote the regeneration and connection of neurons and help to improve the emotional state of patients with depression (Liu et al., 2022; Orlando et al., 2022). This confirms that human pluripotent SC-derived neuronal subtypes have the ability to perform specific circuit repair and functional recovery in the adult brain. In addition to the possibility of differentiating SCs with differentiation function into neuronal cells for transplantation, Guo et al. showed that bone marrow mesenchymal SC-derived exosomes promote hippocampal neuron proliferation and inhibit apoptosis by upregulating the expression of microRNA-26a, which ameliorates hippocampal neuron damage in rats with depression (Guo et al., 2020). This study revealed that SCs ameliorate depression in a miRNA-dependent manner, providing a new perspective for studying the biological mechanism underlying SC therapy for depression.

### 3.4 Effect of SCs on the HPA

The hyperfunction of the HPA is an important physiological change in depression, with increased activity commonly observed in individuals with the condition. Recent studies have highlighted that different types of antidepressants, including MAOIs, TCAs, and SSRIs, lead to the downregulation of HPA activity in both humans and animal models.

To explore the impact of SC transplantation on the HPA-axis in depression, Zhang et al. investigated the antidepressant mechanisms of SCs using mouse models subjected to chronic restraint stress (CRS) and repetitive social failure. Their findings revealed minimal influence of SCs on c-Fos (a marker of neuronal activation) expression in dopamine neurons within the ventral tegmental area (VTA) or on DA concentration in brain homogenates. Furthermore, the researchers examined whether CRS activated CRH neurons in the paraventricular nucleus and found that SCs did not reduce c-Fos expression in CRH neurons or CRH concentration in brain homogenates (Huang et al., 2023). Currently, research on the impact of HPA alterations on depression following SC transplantation is limited. There are no reports addressing the interaction between GC signal transduction and depression-related pathways, such as BDNF, FKBP51, and autophagy. Consequently, confirmation of HPA-axis changes post SC transplantation in depression models remains elusive. Future research should aim to further clarify the relationship between SC transplantation and the HPA in depression, providing a robust theoretical foundation for the application of SCs in the treatment of depression.

## 4 Prospective therapeutic strategies for SCs treatment of depression

Although the safety of traditional antidepressants is generally high, some inevitable side effects such as nausea, insomnia, and headache remain. In addition, about 25% of patients do not respond well to drug treatment. This suggests that existing theories and hypotheses cannot fully explain the pathogenesis of MDD, therefore highlighting the need for more research. Preclinical studies have shown that the combination of neural SCs and antidepressant drugs such as sertraline can reduce depressive-like behaviors in rats with refractory depression (Kigawa et al., 2014). This study provides a theoretical sample for the combination of drugs and SCs for the treatment of depression. However, the biggest challenges of combination therapy are timing of treatment and drug safety, which remain to be confirmed by further studies. In addition, the chronic inflammatory microenvironment in the brain of patients with long-term depression leads to a series of problems such as low survival rate and poor efficacy of transplanted SCs. The development of engineered SCs that can quickly take effect and penetrate the blood-brain barrier will become a new strategy for SC treatment of depression. Therefore, a comprehensive understanding of the pathophysiological mechanism of MDD may greatly improve our understanding of the mechanisms underlying the use of SCs for the treatment of depression and provide important insights into drug combination and SCs engineering.

## 5 Discussion

**FIGURE 1:**
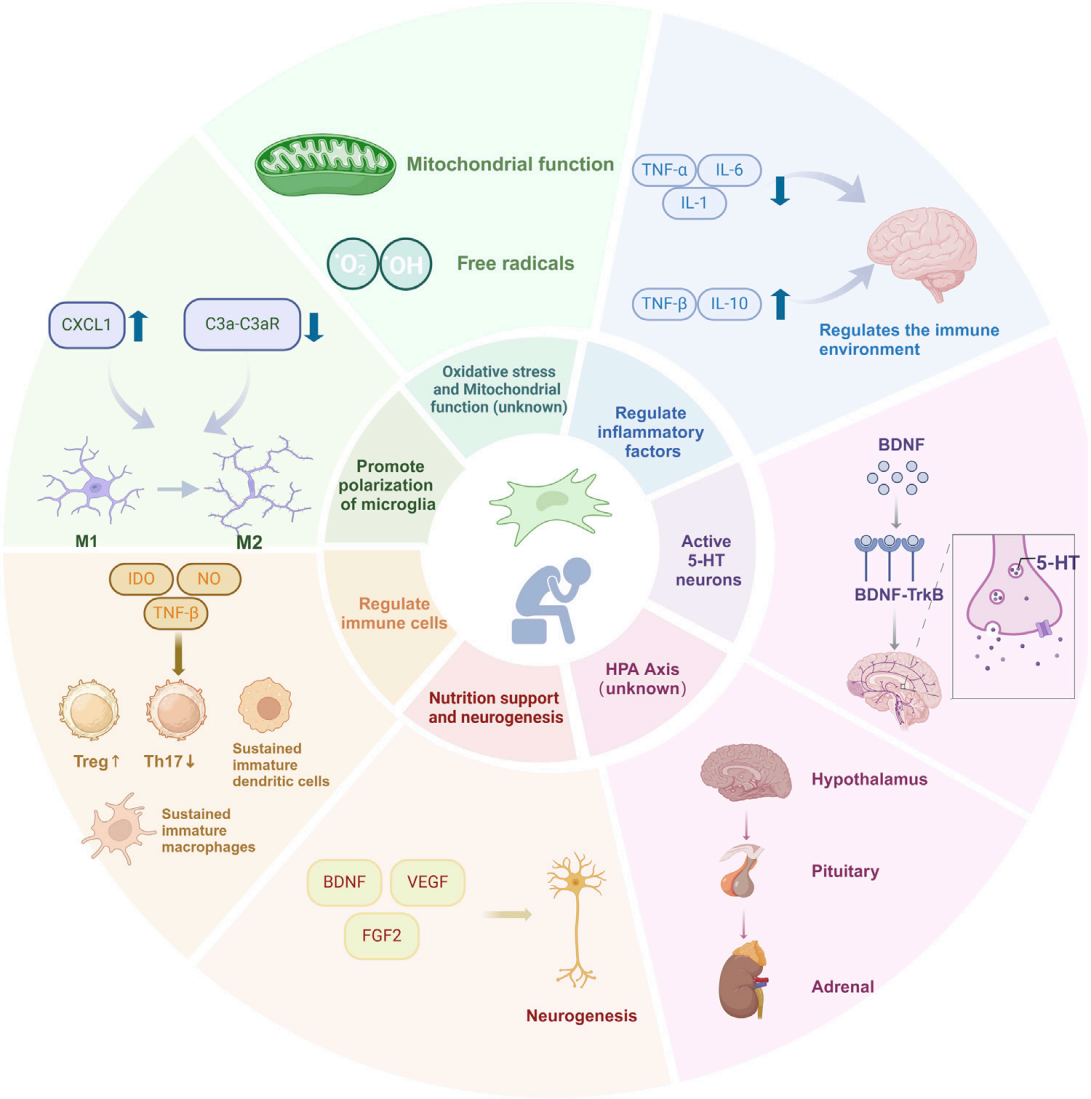
Current understanding of molecular mechanisms underlying SCs therapeutic effects in depression. A multitude of physiological factors and molecular changes that can affect the SCs therapeutic effects in depression. Most factors are further explained under different subheadings in the text.

The pathogenesis of depression is complex, and while traditional treatments can slow down disease progression, their limitations are evident. SC transplantation, as a potential new therapeutic modality, holds promise for promoting the functional restoration of neuronal cells and tissue regeneration, particularly in neurodegenerative diseases and depression. However, the underlying mechanisms remain unclear. As a form of regenerative medicine, SC therapy offers unlimited possibilities for the treatment of many mental disorders. This review provides an in-depth discussion of the possible mechanisms of different sources of SCs for the treatment of depression, and prospectively proposes treatment strategies in order to provide reference for clinical evidence-based drugs (Figure 1).

Experimental data have confirmed that SCs are considered as a potential antidepressant therapy based on their anti-inflammatory and neurotrophic properties. SCs can promote the synthesis and secretion of 5-HT and dopamine to improve the excitability of the brain, so as to play a role in the treatment of depression. Oxidative stress contributes to inflammation and increased production of proinflammatory cytokines, leading to depressive behavior. SCs can improve mitochondrial dysfunction and prevent disordered mitochondria from releasing increased free radicals and other oxidative products to damaged cells. In addition, SCs can trigger anti-inflammatory responses. SC therapy can significantly reduce the expression levels of proinflammatory factors such as IL-6, IL-1, and TNF-α in the serum of patients with depression and upregulate anti-inflammatory factors to improve the brain inflammatory response. In addition, SCs have immunomodulatory properties, which can promote microglia to change from a proinflammatory to an anti-inflammatory state, thereby inhibiting inflammation, playing a neuroprotective role, and improving depressive symptoms. In addition, SCs can promote nerve regeneration through paracrine and nutritional support. And release neurotrophic factors to support the survival of neurons, promote growth and differentiation, and repair damaged nerve tissue. SCs may also improve the symptoms of depression by affecting the activation, proliferation, and differentiation of other immune cells. SCs and their derived exosomes have begun to show potential toward treating depression in clinical trials worldwide. At present, clinical trials of SC products or exosomes for the treatment of depression have been approved in the international platform registration. There are five registered clinical studies involving the use of SCs and the derived exosomes to treat patients with depression in the U.S. clinical trial database (https://clinicaltrials.gov/). Currently, four of these clinical studies (phase 1 and 2) are evaluating the safety, efficacy, and tolerability of SC and exosome administration. As these studies progress, additional results are expected to be published soon, potentially opening new avenues for the treatment of depression. Despite the positive effects of SCs in depression, further studies on their antidepressant mechanisms are needed to determine their suitability for the treatment of depression. Undoubtedly, how to improve the efficacy of SC therapy will be an important trend in future clinical applications. The combination of SCs with other first-line antidepressant drugs and the development of engineered SCs will provide new therapeutic strategies for the treatment of depression.

Although SC therapy provides a new treatment option for patients with depression, it is still at its infancy and the number of preclinical studies is limited. Therefore, SC safety, efficacy, and long-term risks must be thoroughly studied and tested. Although SC therapy has shown potential benefits for the treatment of depression in animal models and preliminary clinical trials, its efficacy and safety in human patients have not been fully validated, and further studies are needed to verify whether SC antidepressant therapy is effective and safe. Furthermore, long-term studies are needed to assess the safety and potential side effects of SC therapy, including the possibility of immune responses, and the risk of cancer. Therefore, to ensure the long-term safety of SC transplantation, clinical trial participants must be carefully monitored for side effects during a long-term follow-up. In addition, the cost and technical threshold of SC therapy are high, and its popularization and large-scale application face great challenges. Finally, ethical and moral issues are unavoidable major challenges in SC therapy. SCs are sourced from sensitive fields such as embryos and umbilical cord blood, therefore resulting in ethical and legal disputes. How to ensure the legality, safety and ethical standards of treatment is a problem that must be solved when promoting SC treatment of depression. Nevertheless, continued research and practice is needed to determine the suitability of SCs for the treatment of depression.

## Glossary

*5-HT*: 5-hydroxytryptamine
*ADSCs*: Adipose-derived MSCs
*BDNF*: Brain-derived neurotrophic factor
*BMSC-Exos*: BMSCs exosomes
*BMSCs*: Bone marrow-derived MSCs
*CNS*: Central nervous system
*CORT*: Corticosterone
*CRH*: Corticotropin-releasing hormone
*CRS*: Chronic restraint stress
*CX3CR1*: C-X3-C motif chemokine receptor 1
*DA*: Dopamine
*DRN*: Dorsal raphe nucleus
*eMSCs*: Encapsulated MSCs
*ESCs*: Embryonic SCs
*Exos*: Exosomes
*GC*: Glucocorticoid
H2S: Hydrogen sulfide
*HPA*: Hypothalamic-pituitary-adrenal axis
*hUC-MSC*: Human umbilical cord blood MSCs
*IDO*: Indoleamine 2,3-dioxygenase
*IL*: Interleukin
*iPSCs*: Induced pluripotent SCs
*LPS*: Lipopolysaccharide
*MAOI*: Monoamine oxidase inhibitors
*MSCs*: Mesenchymal stem cells
*NE*: Norepinephrine
*NTF*: Neurotrophic factors
*ROS*: Reactive oxygen species
*SC*: Stem cell
*SSRIs*: Selective serotonin reuptake inhibitors
TCAs: Tricyclic antidepressants
*Th17*: T helper cell 17
*TLRs*: Toll-like receptors
*TNF-α*: Tumor necrosis factor-α
*VTA*: Ventral tegmental area
